# Reexamining the Impact of Insurance Type on Kidney Transplant Waitlist Status and Posttransplantation Outcomes in the United States After Implementation of the Affordable Care Act

**DOI:** 10.1097/TXD.0000000000001442

**Published:** 2023-01-26

**Authors:** Anna Morenz, James Perkins, André Dick, Bessie Young, Yue-Harn Ng

**Affiliations:** 1 Department of Medicine, University of Washington, Seattle, WA.; 2 Clinical and Bio-Analytics Transplant Laboratory (CBATL), Department of Surgery, University of Washington, Seattle, WA.; 3 Division of Transplantation, Department of Surgery, University of Washington, Seattle, WA.; 4 Division of Transplantation, Department of Surgery, Seattle Children’s Hospital, Seattle, WA.; 5 Division of Nephrology, Department of Medicine, University of Washington, Seattle, WA.

## Abstract

Insurance type, a marker of socioeconomic status, has been associated with poor access to kidney transplant (KT) and worse KT outcomes before the implementation of the Affordable Care Act (ACA) and the revised Kidney Allocation System (KAS). In this study, we assessed if insurance type remained a risk marker for worse waitlist and transplant outcomes after ACA and KAS.

**Methods.:**

Using Scientific Registry of Transplant Recipients data, we assessed insurance type of waitlisted candidates pre- (2008–2014) versus post- (2014–2021) KAS/ACA using chi-square tests. Next, we performed a competing risk analysis to study the effect of private versus public (Medicare, Medicaid, or government-sponsored) insurance on waitlist outcomes and a Cox survival analysis to study posttransplant outcomes while controlling for candidate, and recipient and donor variables, respectively.

**Results.:**

The proportion of overall KT candidates insured by Medicaid increased from pre-KAS/ACA to post-KAS/ACA (from 12 667 [7.3%] to 21 768 [8.8%], *P* < 0.0001). However, KT candidates with public insurance were more likely to have died or become too sick for KT (subdistribution hazard ratio [SHR] = 1.33, confidence interval [CI], 1.30-1.36) or to receive a deceased donor KT (SHR = 1.57, CI, 1.54-1.60) but less likely to receive a living donor KT (SHR = 0.87, CI, 0.85-0.89). Post-KT, KT recipients with public insurance had greater risk of mortality (relative risks = 1.22, CI, 1.15-1.31) and allograft failure (relative risks = 1.10, CI, 1.03-1.29).

**Conclusions.:**

Although the implementation of ACA marginally increased the proportion of waitlisted candidates with Medicaid, publicly insured KT candidates remained at greater risk of being removed from the waitlist, had lower probability of living donor kidney transplantation, and had greater probability of dying post-KT and allograft failure. Concerted efforts to address factors contributing to these inequities in future studies are needed, with the goal of achieving equity in KT for all.

Kidney transplantation (KT) is the preferred treatment option for patients with end-stage kidney disease (ESKD), conferring advantages in terms of patient survival and quality of life compared with remaining on dialysis.^1-3^ Despite extensive research and policy reforms, significant inequities remain in terms of access to KT, as well as KT outcomes, particularly for patients from minoritized racial and ethnic groups and lower socioeconomic status, older patients, and patients with comorbidities.^4^ Type of insurance coverage is one marker for socioeconomic status and social vulnerability that is readily and consistently available to clinicians and transplant programs within the medical record, whereas other measures of socioeconomic status (such as income, education, and wealth) are often not routinely collected or easily accessible to clinicians, transplantation programs, or insurers.

Low socioeconomic status is one important social driver of health that shapes health outcomes by influencing one’s vulnerability to disease (via exposure to environmental or workplace toxins or lack of access to nutrition, transportation, housing stability, etc), as well as one’s access to health care, including medications. In terms of racial and ethnic inequities, Black, Hispanic, and American Indian/Alaska Native patients shoulder a higher burden of negative social drivers of health^5^ and are more likely to be insured by Medicaid or other public insurance than White patients.^6^

The existing literature has demonstrated that concerning inequities exist in the KT care cascade (referral, evaluation, waitlisting, transplantation, and posttransplant) by insurance status. Using the 2005 to 2009 United States Renal Data System data, Johansen et al^7^ demonstrated that patients without private insurance were less likely to be referred for KT assessment. Single-center data revealed that being publicly insured (on Medicaid, Medicare, Veterans’ Affairs, or other government-sponsored insurance) decreased the probability of being waitlisted after referral.^8^ In terms of transplantation, research using 2002–2011 data from the US Scientific Registry of Transplant Recipients (SRTR) found that fewer transplantations than expected were performed among Medicaid beneficiaries for all organs (adult heart, kidney, and lung) except liver.^9^ Similarly, a single-center study found that being publicly insured was associated with a lower probability of any KT, especially living donor KT (LDKT).^10^ Posttransplantation survival was also lower in Medicaid beneficiaries from 2002 to 2011 than in private insurance for all organs.^9^ Based on these findings, DuBay et al^9^ recommended that transplantation programs consider implementing specific posttransplantation care practices to ameliorate decreased survival in the Medicaid population, but no such policies have been enacted to our knowledge.

Since the above publications, 2 major national policy changes aimed at improving healthcare access and equity have been implemented—the Kidney Allocation System (KAS), implemented in December 2014, which credits the start of dialysis as the starting point of waitlisting, hence benefitting minoritized patients who historically have been referred late for KT,^11^ as well as Medicaid expansion (first implemented January 2014 after passage of the Affordable Care Act [ACA]). Since the implementation of the ACA, Harhay et al^12^ showed that there has been increased preemptive waitlisting for KT in Medicaid beneficiaries and an increase in Medicaid coverage among racial and ethnic minorities. No studies have examined whether these significant policy changes have relieved the waitlist and post-KT inequities by insurance status revealed in the above publications, important insights as we near a decade after these policies were enacted and seek to support evidence-based policies moving forward. Using the SRTR data, our study sought to explore the association between insurance type and important KT outcomes, including (1) waitlist status and (2) post-KT outcomes, in the era after major policy changes—KAS and Medicaid expansion.

## MATERIALS AND METHODS

We conducted a retrospective analysis of the SRTR data from December 4, 2008, to December 3, 2014 (pre-KAS/ACA) and from December 4, 2014, to June 30, 2021 (post-KAS/ACA).

### Waitlist Cohort and Outcomes

To assess the waitlist status of candidates in the SRTR data, we analyzed the candidate dataset (from December 4, 2008, to June 30, 2021) and included (1) adult (>17 y old) candidates and (2) US citizens or residents on the waitlist for a kidney only transplant. We excluded candidates who were (1) retransplants or (2) waitlisted for multiorgan transplant (including kidney-pancreas transplants). Insurance status was the major exposure of interest, and we stratified insurance status into public versus private. Public insurance included Medicaid, Medicare, Veterans’ Affairs, or other government-sponsored insurance as defined by a previous study.^10^ All nonpublic insurance was classified as private. In addition, we included candidate age, sex, self-reported race and ethnicity (White, Black, Hispanic, Asian, other—as defined by the United Network of Organ Sharing [UNOS] form), education level (any college education versus none), employment status, body mass index (BMI), cause of kidney disease (diabetes mellitus, glomerulonephritis, hypertension, polycystic kidney disease, other), years on dialysis, and calculated panel reactive antibody.

We first compared the proportion of publicly versus privately insured patients on the KT waitlist pre-KAS/ACA (December 4, 2008–December 3, 2014) to post-KAS/ACA cohort (December 4, 2014–June 30, 2021) to assess trends in insurance coverage over time.

Next, we assessed the waitlist outcomes of KT candidates who have been waitlisted since the implementation of the KAS/ACA (2014–2021) by their insurance status. The main outcome of interest was removal from the waitlist. The following outcomes were treated as competing risks: remaining active on the waitlist and removal from the waitlist because of (1) deceased donor KT (DDKT); (2) LDKT; (3) patient died or was too sick to remain on the waitlist (“died/too sick”); or (4) other. “Other” reasons for waitlist removal included but were not limited to inability to contact the patient and refusal of transplant when offered. No patient who was removed from the waitlist for reasons of being “too sick” or “other” returned to the list during our study period. We adjusted for the candidate variables described above.

### Post-KT Cohort and Outcomes

For our post-KT cohort, we analyzed the KT recipient dataset from 2014 to 2021 and included (1) adult recipients (>17 y old) of DDKT or LDKT and (2) US citizens or residents. We excluded recipients with (1) kidney retransplant or (2) multiple organ transplants. We included the same variables as the candidate data in the recipient dataset with values recorded at the time of the transplant procedure. In addition, we included donor variables that were common to both living and deceased donors (age, gender, race and ethnicity, donation status, history of hypertension, history of diabetes, height, weight, and compatibility via HLA mismatching).

In this analysis, the exposure remained insurance status as described above, which was recorded at the time of transplant. The main outcome was the risk of post-KT patient death or allograft failure.

### Data Source

These data were derived from the SRTR data released September 1, 2021. The UNOS supplied these data as the contractor for the SRTR. The interpretation and reporting of these data are the responsibility of the authors and in no way should be considered an official policy of, or interpretation by, the SRTR or the US Government. The University of Washington Human Subjects Division deems that the SRTR database is deidentified and publicly available and thus not considered human subjects’ data. Therefore, this study was exempt from human subjects’ review.

### Statistical Analysis

Continuous demographic variables were presented as median and the 25% and 75% quartiles. Categorical demographic variables were presented as counts and percentages. Less than 1% of BMI values were missing and imputed with linear regression using age, race, and gender. Any college education and employment status had greater than 2% missing values, and unknown was recorded. All variables were evaluated for significance, and 1 variable was entered into the analysis from variables that demonstrated collinearity to each other. Results were statistically significant if *P* < 0.05.

We performed a competing risk analysis^13^ for the waitlist cohort. The competing risk analysis estimated the univariable and multivariable risks of removal from the waitlist because of insurance status, adjusting for the candidate variables listed above. Competing risks were presented as subdistribution hazard ratios (SHRs) with 95% confidence intervals (CIs). In the post-KT cohort, a Cox model estimated the univariable and multivariable risks of patient death and death-censored graft failure because of insurance status, adjusting for the aforementioned recipient and donor variables. The Cox analyses were presented as relative risks (RRs) with 95% CI. Competing risk analysis was performed using R, version 4.0.0, and the cmprsk 2.2-10 package.^14^ The Cox analysis was performed using the survival package v3.2-7.^15^

## RESULTS

### KT Waitlist Outcomes

**FIGURE 1.**
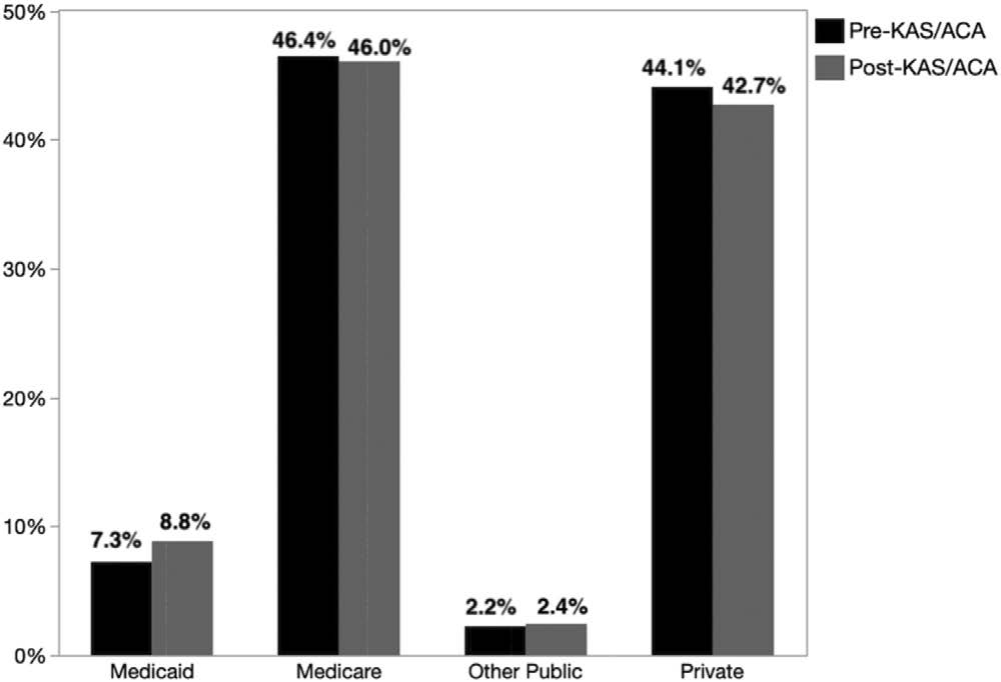
A total of 7.3% of KT candidates (n = 12 667) were insured by Medicaid pre-KAS/ACA, which significantly increased to 8.8% (n = 21 768) of KT candidates insured by Medicaid post-KAS/ACA (*P* < 0.001). Insurance by Medicare decreased from 46.4% (n = 81 188) pre-KAS/ACA to 46.0% (n = 113 347) post-KAS/ACA (*P* < 0.001). ACA, Affordable Care Act; KAS, Kidney Allocation System; KT, kidney transplantation.

A total of 175 335 KT candidates were waitlisted between 2008 and 2021. Comparing the pre- (2008–2014) to post- (2014–2021) KAS/ACA cohort, there was a small but statistically significant increase in the proportion of publicly insured patients on the KT waitlist from 55.9% (n = 97 766) to 57.4% (n = 141 071) (*P* < 0.001). As shown in Figure 1, this was mostly explained by a small but statistically significant increase in Medicaid-covered patients on the KT waitlist from 7.3% (n = 12 667) of all candidates pre-KAS/ACA to 8.8% (n = 21 766) post-KAS/ACA and a small decrease in Medicare-covered patients.

Since the implementation of the KAS/ACA, a total of 247 335 individuals were waitlisted for KT from 2014 to 2021. Of these, 42.6% had private insurance, and 57.4% had public insurance. A majority of waitlisted KT candidates were male (62%), and the median age was 55 y (interquartile range [IQR], 45–63 y). Waitlisted candidates were 40% White, 31% Black, 19% Hispanic, 7.8% Asian, and 2.5% other. Fifty-one percent of waitlisted candidates had at least some college education, and 46% were employed.

Table 1 demonstrates baseline characteristics and outcomes for KT candidates stratified by insurance status (private versus public). KT candidates with public insurance were more likely to be older, female, non-White, and unemployed; have less education; have kidney disease due to diabetes or hypertension; have comorbid diabetes; and have higher dialysis vintage.

**TABLE 1.** Baseline characteristics and outcomes of waitlist candidates stratified by insurance status

Characteristic	Overall, N = 247 335	Private, n = 105 360	Public, n = 141 975	*P*
Age, y	55 (45, 63)	53 (44, 60)	56 (45, 65)	<0.001
Female	94 963 (38%)	40 061 (38%)	54 902 (39%)	0.001
Race and ethnicity				<0.001
White	97 752 (40%)	49 021 (47%)	48 731 (34%)	
Black	76 488 (31%)	27 173 (26%)	49 315 (35%)	
Hispanic	47 515 (19%)	17 123 (16%)	30 392 (21%)	
Asian	19 370 (7.8%)	9632 (9.1%)	9738 (6.9%)	
Other	6210 (2.5%)	2411 (2.3%)	3799 (2.7%)	
College education				<0.001
Yes	127 232 (51%)	64 131 (61%)	63 101 (44%)	
None	111 952 (45%)	38 288 (36%)	73 664 (52%)	
Unknown	8151 (3.3%)	2941 (2.8%)	5210 (3.7%)	
Employment				<0.001
Yes	82 947 (34%)	60 379 (57%)	22 568 (16%)	
No	157 171 (64%)	42 193 (40%)	114 978 (81%)	
Unknown	7217 (2.9%)	2788 (2.6%)	4429 (3.1%)	
BMI (kg/m^2^)				<0.001
<18.5	2744 (1.1%)	1166 (1.1%)	1578 (1.1%)	
18.5–24.9	54 232 (22%)	22 617 (21%)	31 615 (22%)	
25–29.9	80 949 (33%)	33 731 (32%)	47 218 (33%)	
30–39.9	102 366 (41%)	44 655 (42%)	57 711 (41%)	
≥40	7044 (2.8%)	3191 (3.0%)	3853 (2.7%)	
Kidney disease diagnosis				<0.001
Diabetes mellitus	96 681 (39%)	36 588 (35%)	60 093 (42%)	
HTN	55 526 (22%)	20 404 (19%)	35 122 (25%)	
GN	41 065 (17%)	21 594 (20%)	19 471 (14%)	
PKD	18 010 (7.3%)	11 146 (11%)	6864 (4.8%)	
Congenital/hereditary	3801 (1.5%)	2109 (2.0%)	1692 (1.2%)	
Other	32 252 (13%)	13 519 (13%)	18 733 (13%)	
Comorbidity				
Diabetes mellitus	115 647 (47%)	44 028 (42%)	71 619 (50%)	<0.001
Years on dialysis				<0.001
Not on dialysis	57 531 (23%)	35 037 (33%)	22 494 (16%)	
>0–1 y	12 909 (5.2%)	7540 (7.2%)	5369 (3.8%)	
1–5 y	103 025 (42%)	41 855 (40%)	61 170 (43%)	
>5 y	73 870 (30%)	20 928 (20%)	52 942 (37%)	
CPRA	0 (0, 17)	0 (0, 11)	0 (0, 19)	<0.001
Outcome				
Waitlist status				<0.001
Active	71 233 (29%)	32 226 (31%)	39 007 (27%)	
Died/too sick	44 984 (18%)	16 115 (15%)	28 869 (20%)	
DDKT	74 447 (30%)	27 173 (26%)	47 274 (33%)	
LDKT	32 016 (13%)	20 992 (20%)	11 024 (7.8%)	
Other	24 655 (10.0%)	8854 (8.4%)	15 801 (11%)	

BMI, body mass index; CPRA, calculated panel reactive antibodies; DDKT, deceased donor kidney transplant; GN, glomerulonephritis; HTN, hypertension; LDKT, living donor kidney transplant; PKD, polycystic kidney disease.

**FIGURE 2.**
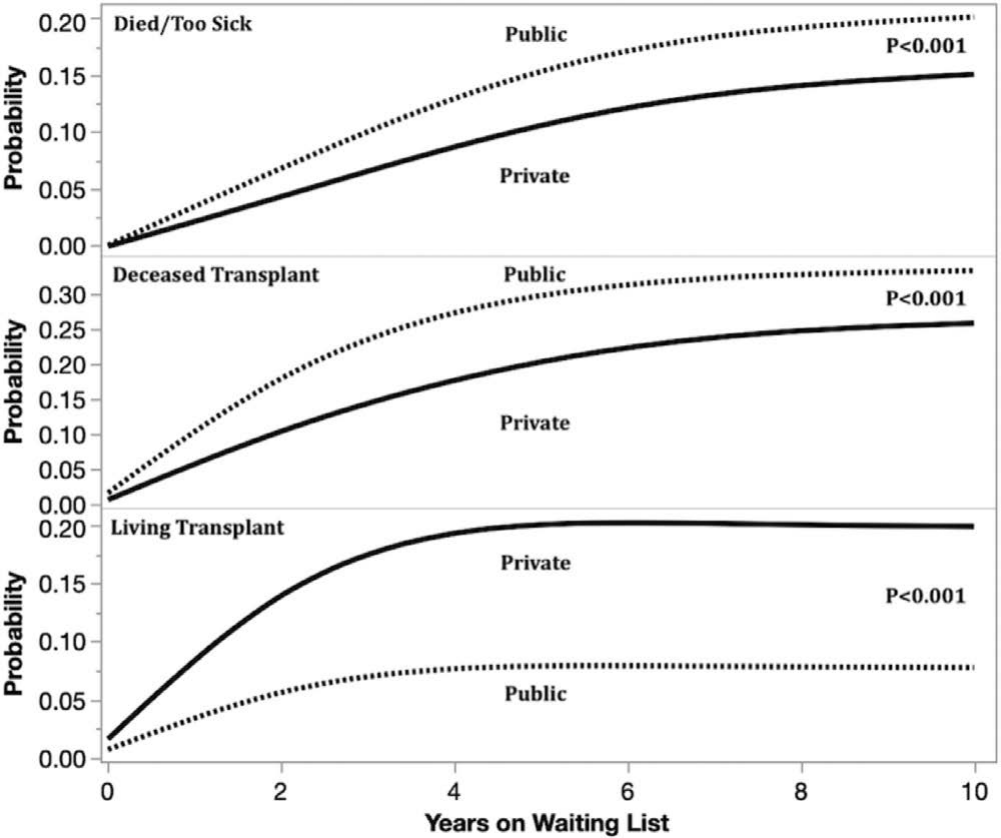
After the KAS revision and Medicaid expansion under the ACA, publicly insured KT candidates were significantly more likely to be removed from the waitlist because of death or becoming too sick or for DDKT than privately insured candidates. They were less likely to receive an LDKT than privately insured candidates. ACA, Affordable Care Act; DDKT, deceased donor kidney transplantation; KAS, Kidney Allocation System; KT, kidney transplantation; LDKT, living donor kidney transplantation.

As shown in Figure 2, KT candidates with public insurance were more likely to be removed from the waitlist because of reasons of “died/become too sick for KT” (SHR = 1.33, CI, 1.30-1.36) or “other” (SHR = 1.44, CI, 1.40-1.49) than privately insured patients. Within this category of “other,” KT candidates with public insurance were significantly more likely to have been removed from the waitlist because of refusal of KT offer or being unable to contact them (*P* < 0.001; data not shown). In this cohort, KT candidates with public insurance were more likely to receive a DDKT (SHR = 1.57, CI, 1.54-1.60) but less likely to receive an LDKT (SHR = 0.87, CI, 0.85-0.89). Additional variables significantly associated with waitlist removal are shown in **Table S1, SDC**, http://links.lww.com/TXD/A492.

### Post-KT Outcomes

A total of 105 346 individuals received an LDKT or a DDKT during our study period. Of these, 7952 (7.5%) died, whereas 5451 (5.2%) experienced allograft failure. Thirty-one percent had private insurance, and a majority (69%) had public insurance. A majority of transplanted individuals were male (61%), and the median age was 55 y (IQR, 44-64 y). Transplanted individuals were 44% White, 29% Black, 18% Hispanic, 7.3% Asian, and 2.3% other. Fifty-five percent had at least some college education, and 39% were employed.

Table 2 demonstrates baseline characteristics for KT recipients stratified by private versus public insurance. KT recipients with public insurance were more likely to be older, male, non-White, and unemployed; have less education; have a higher BMI, have kidney disease due to diabetes or hypertension; and have higher dialysis vintage.

**TABLE 2.** Baseline characteristics of kidney transplant recipients, stratified by insurance status

Characteristic	Overall, N = 105 346	Private, n = 32 677^*a*^	Public, n = 72 669^*a*^	*P* ^*b*^
Recipient				
Age	55 (44, 64)	51 (41, 60)	56 (45, 65)	<0.001
Female	40 966 (39%)	12 962 (40%)	28 004 (39%)	<0.001
Race and ethnicity				<0.001
White	45 977 (44%)	18 696 (57%)	27 281 (38%)	
Black	30 534 (29%)	5668 (17%)	24 866 (34%)	
Hispanic	18 752 (18%)	5070 (16%)	13 682 (19%)	
Asian	7669 (7.3%)	2610 (8.0%)	5059 (7.0%)	
Other	2414 (2.3%)	633 (1.9%)	1781 (2.5%)	
College education				<0.001
Yes	57 783 (55%)	21 523 (66%)	36 260 (50%)	
None	45 625 (43%)	10 545 (32%)	35 080 (48%)	
Unknown	1938 (1.8%)	609 (1.9%)	1329 (1.8%)	
Employment				<0.001
Yes	40 950 (39%)	21 680 (66%)	19 270 (27%)	
No	62 109 (59%)	10 217 (31%)	51 892 (71%)	
Unknown	2287 (2.2%)	780 (2.4%)	1507 (2.1%)	
BMI (kg/m^2^)				<0.001
<18.5	1603 (1.5%)	525 (1.6%)	1078 (1.5%)	
18.5–24.9	27 664 (26%)	9115 (28%)	18 549 (26%)	
25–29.9	35 512 (34%)	11 004 (34%)	24 508 (34%)	
30.0–39.9	38 566 (37%)	11 487 (35%)	27 079 (37%)	
≥40	2001 (1.9%)	546 (1.7%)	1455 (2.0%)	
Kidney diagnosis				<0.001
Congenital/hereditary	2379 (2.3%)	1109 (3.4%)	1270 (1.7%)	
Diabetes mellitus	31 154 (30%)	7285 (22%)	23 869 (33%)	
GN	22 848 (22%)	9099 (28%)	13 749 (19%)	
HTN	24 502 (23%)	5543 (17%)	18 959 (26%)	
Other	14 127 (13%)	4545 (14%)	9582 (13%)	
PKD	10 336 (9.8%)	5096 (16%)	5240 (7.2%)	
Years on dialysis				<0.001
Not on dialysis	18 147 (17%)	11 594 (35%)	6553 (9.0%)	
>0–1	13 493 (13%)	7032 (22%)	6461 (8.9%)	
1–5	43 833 (42%)	11 216 (34%)	32 617 (45%)	
>5	29 873 (28%)	2835 (8.7%)	27 038 (37%)	
CPRA	0 (0, 16)	0 (0, 9)	0 (0, 17)	<0.001
Donor				
Age	41 (30, 52)	41 (30, 52)	41 (30, 53)	0.006
Female	48 810 (46%)	16 785 (51%)	32 025 (44%)	<0.001
Race and ethnicity				<0.001
White	72 394 (69%)	23 497 (72%)	48 897 (67%)	
Black	12 857 (12%)	2967 (9.1%)	9890 (14%)	
Hispanic	14 867 (14%)	4375 (13%)	10 492 (14%)	
Asian	3255 (3.1%)	1218 (3.7%)	2037 (2.8%)	
Other	1973 (1.9%)	620 (1.9%)	1353 (1.9%)	
Donation status				<0.001
Living donor	31 697 (30%)	17 590 (54%)	14 107 (19%)	
Anoxia	33 080 (31%)	7051 (22%)	26 029 (36%)	
CVA	17 704 (17%)	3120 (9.5%)	14 584 (20%)	
Other	2382 (2.3%)	484 (1.5%)	1898 (2.6%)	
Trauma	20 483 (19%)	4432 (14%)	16 051 (22%)	
History of hypertension				<0.001
Yes	23 252 (22%)	4665 (14%)	18 587 (26%)	
No	81 152 (77%)	27 762 (85%)	53 390 (73%)	
Unknown	942 (0.9%)	250 (0.8%)	692 (1.0%)	
History of diabetes mellitus				<0.001
Yes	5903 (5.6%)	1067 (3.3%)	4836 (6.7%)	
No	98 590 (94%)	31 379 (96%)	67 211 (92%)	
Unknown	853 (0.8%)	231 (0.7%)	622 (0.9%)	
Height, cm	170 (163, 178)	170 (163, 178)	170 (163, 178)	<0.001
Weight, kg	79 (67, 93)	78 (67, 90)	80 (67, 94)	<0.001
Transplant				
ABO matching				<0.001
Compatible	9499 (9.0%)	4841 (15%)	4658 (6.4%)	
Identical	94 004 (89%)	27 245 (83%)	66 759 (92%)	
Incompatible	1843 (1.7%)	591 (1.8%)	1252 (1.7%)	
HLA A mismatch				<0.001
1	44 075 (42%)	14 470 (44%)	29 605 (41%)	
2	47 657 (45%)	12 796 (39%)	34 861 (48%)	
Unknown	625 (0.6%)	382 (1.2%)	243 (0.3%)	
0	12 989 (12%)	5029 (15%)	7960 (11%)	
HLA B mismatch				<0.001
1	31 500 (30%)	11 118 (34%)	20 382 (28%)	
2	65 305 (62%)	17 907 (55%)	47 398 (65%)	
Unknown	626 (0.6%)	383 (1.2%)	243 (0.3%)	
0	7915 (7.5%)	3269 (10%)	4646 (6.4%)	
HLA DR mismatch				<0.001
1	51 315 (49%)	15 506 (47%)	35 809 (49%)	
2	37 496 (36%)	11 261 (34%)	26 235 (36%)	
Unknown	625 (0.6%)	382 (1.2%)	243 (0.3%)	
0	15 910 (15%)	5528 (17%)	10 382 (14%)	

^*a*^Statistics presented: median (interquartile range); n (%).

^*b*^Statistical tests performed: Wilcoxon rank-sum test; chi-square test of independence.

BMI, body mass index; CPRA, calculated panel reactive antibodies; GN, glomerulonephritis; HTN, hypertension; KDPI, kidney donor profile index; PKD, polycystic kidney disease.

**FIGURE 3.**
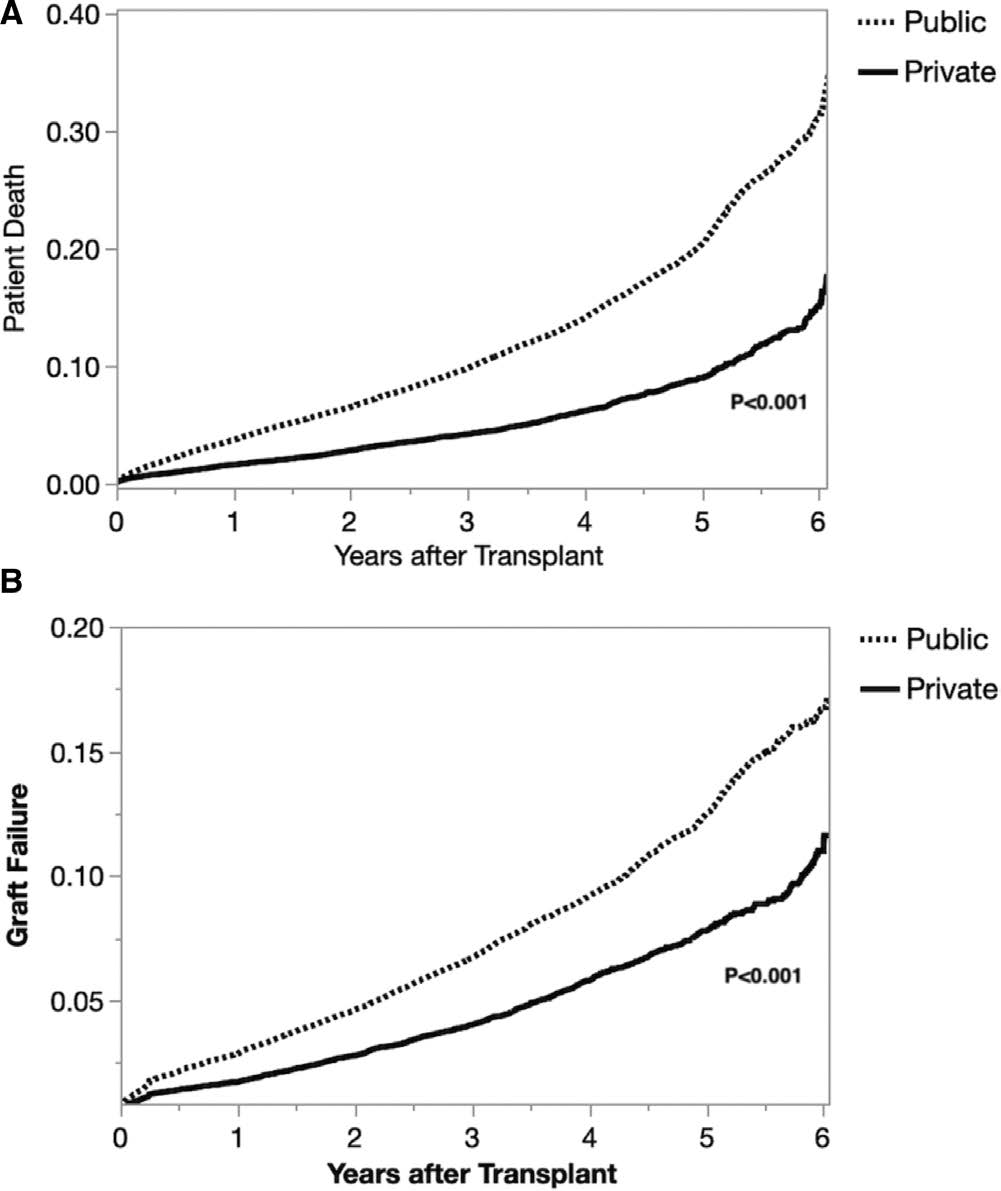
Likelihood of patient death and allograft failure after KT are demonstrated, stratified by insurance status. A, After the KAS revision and Medicaid expansion under the ACA, publicly insured KT recipients were significantly more likely to die than privately insured recipients (9.2% vs 4.0%, respectively; RR = 1.22, CI, 1.15-1.31). B, After the KAS revision and Medicaid expansion under the ACA, publicly insured KT recipients were significantly more likely to suffer allograft failure than privately insured recipients (5.8% vs 3.7%, respectively; RR = 1.10, CI, 1.03-1.29). ACA, Affordable Care Act; CI, confidence interval; KAS, Kidney Allocation System; KT, kidney transplantation; RR, relative risks.

For post-KT outcomes, 9.2% of publicly insured KT recipients died compared with 4.0% of privately insured recipients. Thus, as shown in Figure 3A, KT recipients with public insurance had a 22% higher probability of mortality post-KT (RR = 1.22, CI, 1.15-1.31) than those with private insurance. In terms of allograft failure, 5.8% of publicly insured KT recipients experienced allograft failure compared with 3.7% of privately insured recipients. As shown in Figure 3B, there was a 10% higher probability of allograft failure after KT for publicly insured KT recipients than for privately insured recipients (RR = 1.10, CI, 1.03-1.29). Additional variables significantly associated with post-KT outcomes are shown in **Table S2, SDC**, http://links.lww.com/TXD/A492.

## DISCUSSION

In our study, we found that, in the 7-y period after implementation of Medicaid expansion under the ACA and the 2014 KAS, there was a small but statistically significant increase in the proportion of KT candidates on the waitlist covered by Medicaid from 7.3% to 8.8% relative to Medicare (46.3%–45.8%). This aligns with overall trends demonstrating that coverage increases have been driven by the enrollment of adults made eligible for Medicaid in states that opted to expand their Medicaid programs under the ACA.^16^ As expected, the proportion of publicly insured individuals increased from pre- to posttransplant because of the end-stage renal disease Medicare entitlement.

Unfortunately, however, the coverage gains for KT candidates—and other provisions of the ACA and KAS—have not translated into improved outcomes. Insurance type continues to negatively impact KT waitlist and posttransplant outcomes. KT candidates with public insurance were still significantly more likely to be removed from the waitlist for dying or becoming too sick and for other reasons (such as refusal or unable to contact). Publicly insured individuals were more likely to receive a DDKT but less likely to receive an LDKT. We also found that KT recipients with public insurance had a higher risk of posttransplant mortality and allograft failure, a post-KT outcome that has not been previously linked to insurance type. The increased likelihood of receiving a DDKT may in part reflect the intended impact of the KAS policy in terms of counting total time on dialysis toward KT wait time because publicly insured KT candidates are more likely to have higher dialysis vintage and experience delayed referral for KT. Although at first glance this finding of increased likelihood of DDKT for publicly insured KT candidates may appear a positive step forward, it likely also reflects the lower probability of publicly insured KT candidates receiving an LDKT (7.8% of KT candidates) than privately insured candidates (20%). In sum, 46% of privately insured KT candidates on the waitlist received a transplant (DDKT or LDKT) compared with 40.8% of publicly insured KT candidates, a notable inequity.

A particularly salient and concerning finding from our analysis is that publicly insured KT candidates were more likely than privately insured candidates to have been removed from the waitlist for other reasons, including but not limited to refusal and inability to contact. This reflects a need for targeted education interventions and intensification of resources to support publicly insured individuals on the waitlist to ensure they remain active on the waitlist and are ultimately transplanted. The current multidisciplinary approach used by transplant centers is clearly inadequate to keep these patients on the waitlist, suggesting that more assertive interventions such as the use of peer navigators,^17^ culturally sensitive interventions,^18^ and systemic approaches to address systemic racism^19^ are required.

Our study found that, despite advances in overall healthcare access and decreasing mortality under the ACA, KT recipients with public insurance remained at significantly greater risk of posttransplant mortality and allograft failure. Although additional research is needed to explain this finding, we hypothesize that the overall well-being of a KT recipient relies not only on the adherence to post-KT follow-up and immunosuppressive medications but also on non-KT health components (such as continuity with primary care, other comorbid health conditions, and unaddressed social drivers of health), all of which may be playing a complex role in contributing to an increased risk of mortality and allograft failure. These same factors may also play a role in publicly insured KT candidates’ higher risk of dying or becoming too sick while on the waitlist.

An important limitation of this study was that only candidates already waitlisted for KT were included, which does not address barriers related to KT referral or time to waitlisting, both of which are crucial barriers for a majority of patients with ESKD. As shown by previous single-centered studies, patients with public insurance have a lower probability of waitlisting^8^; hence any intervention planned should be expanded to all patients with ESKD with strong collaboration between transplant centers, dialysis units, and community nephrologists. Another limitation was the lack of information regarding income in the SRTR database, which prevented direct comparison between insurance type and income. More studies will be required to assess the interaction between the different social drivers of health on KT outcomes to better understand the mechanisms and potential causal pathways linking public insurance status to adverse KT waitlist and posttransplant outcomes. Future multicentered trials will be required to address whether intervening on different social drivers of health has synergistic effects in affecting KT waitlisting or outcomes. Using the SRTR as our data source, we acknowledge that there are limitations inherent to large data analyses, including missing data and the inability to detect nuanced situations, such as individual changes in patients. For example, patients may change insurance throughout the transplant process, but this is not well captured in the SRTR database and thus not captured in our analysis. Finally, we acknowledge that the race category in the United Network for Organ Sharing database conflates race and ethnicity without clearly mutually exclusive categories, an issue that has been rectified by UNOS beginning in January 2022 but not for the timeframe of our data of interest.^20^ Strengths of this study include the large sample size available through the SRTR database, as well as the relative completeness of the data. Another strength is the recency of data, which allows us to address the impact of current policies on KT outcomes and thus inform current policymaking deliberations.

In conclusion, public insurance can be used as a readily available risk marker for transplant programs to identify patients in need of greater levels of support on the KT waitlist and posttransplant, as well as to develop closer linkages to primary care for these patients who may primarily see a nephrologist. Qualitative data would be valuable in understanding what kinds of specific interventions would be viewed as beneficial by publicly insured KT candidates or recipients. This is a reminder that, to achieve equitable health outcomes, transplantation programs need to be flexible in meeting individual patients where they are and addressing unique barriers to accessing care that they may face, rather than providing a standard, one-size-fits-all approach to accessing care.

From the standpoint of insurers and health systems, state Medicaid agencies and Medicare could incentivize health systems to track and improve posttransplantation mortality rates among these socioeconomically marginalized populations with careful attention to avoid unintended consequences (analogous to Medicaid initiatives to improve maternal mortality, which include innovative payment and delivery models).^21^ However, as noted by Dick et al in similar work involving the pediatric liver transplant population, it is important to remember the limitations of healthcare systems in mitigating the social drivers of health, which require structural changes reliant on political advocacy, community organizing, and action (such as increasing minimum wage, access to affordable housing, access to education, etc).^22^ Just as we expect evidence-based treatments for individual diseases, we need evidence-based policies and solutions to systemic health inequities. Physician and patient voices, often via collective organizations, can be valuable and influential in this realm.

There is growing recognition that excellent medical care alone cannot address what makes people sick or improve their health. We often fail to assess and address the upstream factors that significantly impact our point of care outcomes. To create meaningful and sustainable changes we will need advocacy and policy changes at all levels to create health equity and deliver the highest quality care.

## Supplementary Material


