# Supplementary material for: Reexamining the Impact of Insurance Type on Kidney Transplant Waitlist Status and Posttransplantation Outcomes in the United States After Implementation of the Affordable Care Act
Source: Transplant Direct. 2023 Jan 26;9(2):e1442. doi: 10.1097/TXD.0000000000001442 (PMC9891441; doi:10.1097/TXD.0000000000001442)
Supplement: Supplementary file 1 [file txd-9-e1442-s001.pdf]

SUPPLEMENTAL TABLES:

Table S1. Competing risk analysis for kidney transplant waitlist outcomes

Variables	Died or Too Sick	P	Deceased Tx	P	Living Tx	P	Other[a]	P
Public Insurance	1.33 (1.30-1.36)[b]	**<0.001**	1.57 (1.54-1.60)	**<0.001**	0.87 (0.85-0.89)	**<0.001**	1.44 (1.40-1.49)	**<0.001**
Age[c]	1.40 (1.39-1.41)	**<0.001**	0.96 (0.95-0.97)	**<0.001**	0.81 (0.80-0.82)	**<0.001**	1.04 (1.03-1.05)	**<0.001**
Female Gender	0.88 (0.86-0.89)	**<0.001**	0.97 (0.96-0.99)	**<0.001**	0.92 (0.90-0.94)	**<0.001**	1.06 (1.03-1.09)	**<0.001**
**Race and Ethnicity**								
White	Ref							
Black	0.89 (0.86-0.91)	**<0.001**	1.09 (1.07-1.11)	**<0.001**	0.42 (0.41-0.44)	**<0.001**	1.07 (1.04-1.11)	**<0.001**
Hispanic	0.80 (0.78-0.82)	**<0.001**	0.94 (0.92-0.96)	**<0.001**	0.79 (0.76-0.81)	**<0.001**	0.92 (0.89-0.96)	**<0.001**
Asian	0.64 (0.62-0.67)	**<0.001**	0.90 (0.87-0.92)	**<0.001**	0.51 (0.49-0.54)	**<0.001**	0.66 (0.62-0.69)	**<0.001**
Other	0.87 (0.82-0.93)	**<0.001**	1.06 (1.01-1.11)	**0.02**	0.59 (0.54-0.64)	**<0.001**	1.17 (1.08-1.27)	**<0.001**
**College Education**								
Yes	Ref							
None	0.94 (0.92-0.96)	**<0.001**	0.92 (0.91-0.94)	**<0.001**	0.72 (0.70-0.74)	**<0.001**	1.08 (1.05-1.11)	**<0.001**
Unknown	1.05 (1.00-1.10)	**0.046**	0.49 (0.46-0.52)	**<0.001**	0.69 (0.64-0.75)	**<0.001**	1.19 (1.11-1.27)	**<0.001**
**Employment**								
Yes	Ref							
No	1.36 (1.33-1.40)	**<0.001**	0.92 (0.90-0.94)	**<0.001**	0.80 (0.78-0.82)	**<0.001**	1.31 (1.27-1.35)	**<0.001**
Unknown	0.89 (0.84-0.95)	**<0.001**	0.64 (0.61-0.68)	**<0.001**	0.82 (0.76-0.89)	**<0.001**	0.70 (0.65-0.76)	**<0.001**
**BMI[d]**								
<18.5	1.29 (1.17-1.43)	**<0.001**	0.95 (0.89-1.02)	0.17	1.06 (0.97-1.16)	0.18	1.02 (0.90-1.15)	0.79

18.5 to 24.9	Ref							
25.0 to 29.9	0.92 (0.89-0.95)	**<0.001**	0.98(0.96-0.99)	**0.04**	0.92 (0.89-0.95)	**<0.001**	0.91 (0.88-0.94)	**<0.001**
30.0 to 39.9	0.93 (0.91-0.95)	**<0.001**	0.93 (0.92-0.95)	**<0.001**	0.79 (0.77-0.82)	**<0.001**	0.95 (0.92-0.99)	**0.007**
≥40	0.89 (0.84-0.95)	**<0.001**	0.62 (0.59-0.65)	**<0.001**	0.60 (0.55-0.65)	**<0.001**	1.31 (1.23-1.39)	**<0.001**
**Diagnosis**								
GN	Ref							
Diabetes	1.94 (1.87-2.01)	**<0.001**	0.69 (0.67-0.70)	**<0.001**	0.65 (0.63-0.67)	**<0.001**	1.27 (1.22-1.33)	**<0.001**
Hypertension	1.21(1.16-1.25)	**<0.001**	0.90 (0.88-0.92)	**<0.001**	0.78 (0.75-0.81)	**<0.001**	1.26 (1.21-1.32)	**<0.001**
PKD	0.82 (0.77-0.86)	**<0.001**	1.07 (1.04-1.10)	**<0.001**	0.99 (0.95-1.02)	0.48	0.95 (0.89-1.02)	0.18
Congenital/ Hereditary	0.87 (0.77-0.99)	**0.03**	1.07 (1.01-1.14)	**0.02**	0.84 (0.79-0.90)	**<0.001**	1.13 (1.01-1.28)	**0.04**
Other	1.14 (1.09-1.19)	**<0.001**	0.86 (0.84-0.88)	**<0.001**	0.70 (0.68-0.73)	**<0.001**	1.14 (1.08-1.20)	**<0.001**
**Years on dialysis**								
0	Ref							
>0 to 1	2.83 (2.63-3.05)	**<0.001**	3.07 (2.95 -3.21)	**<0.001**	2.92(2.82-3.02)	**<0.001**	2.12 (1.92-2.34)	**<0.001**
1 to 5	2.79 (2.71-2.88)	**<0.001**	2.05 (2.01-2.10)	**<0.001**	0.50(0.48-0.51)	**<0.001**	1.67 (1.61-1.74)	**<0.001**
>5	0.84 (0.82-0.87)	**<0.001**	0.96 (0.93-0.98)	**<0.001**	0.06(0.05-0.07)	**<0.001**	0.53 (0.51-0.55)	**<0.001**
CPRA by 100	1.04 (1.01-1.07)	**0.03**	1.13 (1.10-1.16)	**<0.001**	0.43(0.41-045)	**<0.001**	0.80 (0.76-0.83)	**<0.001**

[a]Includes refused transplant and unable to contact candidate
[b]SHR with 95% Confidence Interval
[c]For every increase in 10 years
[d]Abbreviations in order shown: BMI (body mass index), HTN (hypertension), GN (glomerulonephritis), PKD (polycystic kidney disease), CPRA (calculated panel reactive antibodies)

Table S2. Multivariable Cox models for death and death-censored allograft failure using donor variables common to both living and deceased donors

Variables	Death	P	Allograft Failure	P
**Recipient Variables**				
Public insurance at transplantation	1.22(1.15-1.31)[a]	**<0.001**	1.10(1.03-1.29)	0.009
Age[b]	1.57(1.54-1.61)	**<0.001**	0.83(0.81-0.84)	**<0.001**
Female gender	0.83(0.79-0.88)	**<0.001**	0.93(0.88-0.98)	**0.01**
Race and Ethnicity				
White	Ref		Ref	
Black	0.86(0.82-0.91)	**<0.001**	1.29(1.21-1.38)	**<0.001**
Hispanic	0.81(0.75-0.86)	**<0.001**	0.84(0.78-0.92)	**<0.001**
Asian	0.62(0.56-0.69)	**<0.001**	0.70(0.61-0.79)	**<0.001**
Other				
College education				
None	Ref		Ref	
Yes	0.95(0.91-0.99)	**0.03**		
Unknown				
Employment				
No	Ref		Ref	
Yes	0.82(0.78-0.87)	**<0.001**	1.86(0.80-0.91)	**<0.001**
Unknown				
BMI[c]				
<18.5				
18.5 to 24.9	Ref		Ref	
25.0 to 29.9				
30.0 to 39.9	1.01(1.05-1.15)	**<0.001**	1.21(1.14-1.28)	**<0.001**
≥40	1.55(1.33-1.81)	**<0.001**	1.32(1.10-1.58)	**0.003**
Kidney disease diagnosis				
GN	Ref		Ref	
Diabetes	1.87(1.73-2.01)	**<0.001**	0.92(0.86-0.99)	**0.02**
HTN	1.19(1.10-1.30)	**<0.001**	0.91(0.85-0.98)	**0.01**
PKD	0.88(0.78-0.98)	**0.02**	0.73(0.64-0.82)	**<0.001**
Congenital/Hereditary				
Other	1.21(1.11-1.33)	**<0.001**		
Years on dialysis				
0	Ref		Ref	
>0 to 1	1.22(1.10-1.36)	**<0.001**	1.17(1.03-1.32)	**0.01**
1 to 5	1.54(1.41-1.68)	**<0.001**	1.29(1.17-1.44)	**<0.001**
>5	1.97(1.80-2.17)	**<0.001**	1.30(1.16-1.45)	**<0.001**

CPRA by 10	1.01(1.003-1.02)	**0.006**		
**Donor Variables**				
Age[b]	1.01(1.005-1.02)	**<0.001**	1.18(1.15-1.21)	**<0.001**
Female gender				
Race and Ethnicity				
White	Ref		Ref	
Black	1.12(1.05-1.20)	**<0.001**	1.24(1.16-1.34)	**<0.001**
Hispanic				
Asian				
Other				
Donation Status				
Trauma	Ref		Ref	
Anoxia				
CVA			1.12(1.04-1.20)	**0.002**
Alive	0.63(0.59-0.68)	**<0.001**	0.55(0.51-0.61)	**<0.001**
Other			1.28(1.10-1.49)	**0.001**
History of HTN				
No	Ref		Ref	
Yes	1.09(1.04-1.16)	**<0.001**	1.20(1.12-1.29)	**<0.001**
Unknown				
History of diabetes				
No	Ref		Ref	
Yes	1.33(1.05-1.23)	**0.002**	1.51(1.37-1.66)	**<0.001**
Unknown				
Height (cm) by 10 cm	0.97(0.96-0.99)	**<0.001**	0.90(0.88-0.91)	**<0.001**
Weight (lb) by 10 lbs				
**Matching**				
ABO Matching				
Identical	Ref		Ref	
Compatible				
Incompatible				
HLA A Mismatch				
Zero	Ref		Ref	
One				
Two				
Unknown				
HLA B Mismatch				
Zero	Ref		Ref	
One				
Two				

Unknown				
HLA DR Mismatch				
Zero	Ref		Ref	
One	1.07(1.01-1.15)	**0.04**	1.15(1.06-1.25)	**0.001**
Two	1.12(1.04-1.20)	**0.002**	1.22(1.12-1.33)	**<0.001**
Unknown				

In the multivariable analysis, only the significant relative risk (RR) and confidence intervals (CI) were recorded.

[a]RR with 95% CI

[b]For every increase in 10 years

[c]Abbreviations in order shown: BMI (body mass index), GN (glomerulonephritis), HTN (hypertension), PKD (polycystic kidney disease), CPRA (calculated panel reactive antibodies), KDPI (kidney donor profile index)
